# Supplementary material for: Evidence for a task-dependent switch in subthalamo-nigral basal ganglia signaling
Source: Nat Commun. 2017 Oct 19;8:1039. doi: 10.1038/s41467-017-01023-3 (PMC5715140; doi:10.1038/s41467-017-01023-3)
Supplement: Supplementary file 1 — Supplementary Information [file 41467_2017_1023_MOESM1_ESM.pdf]

Supplementary Material
I. Figures

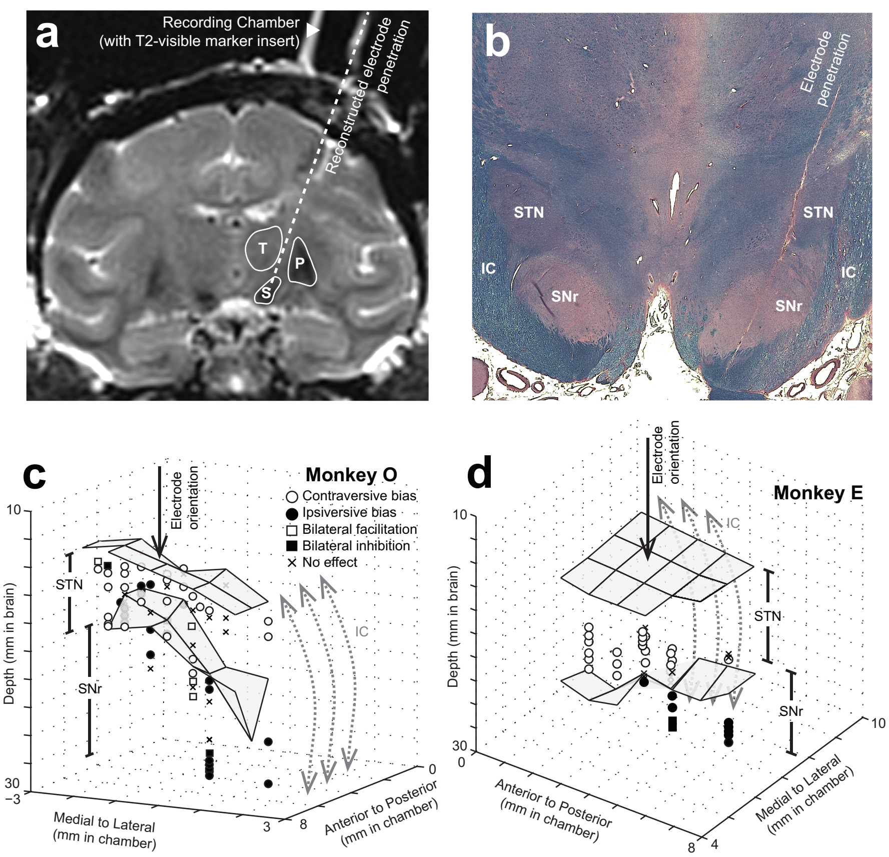

Supplementary Figure 1. Anatomical localization and electrophysiological mapping of the subthalamic nucleus (STN) and substantia nigra pars reticulata (SNr).

(a) MRI coronal plane. STN and SNr were localized anatomically in monkey O by T2-weighted turbo spin-echo (TSE). Electrode trajectories roughly followed the internal capsule. T, thalamus; S, subthalamic nucleus and substantia nigra; P, pallidum. **(b)** Histology, coronal slice. STN and SNr penetrations were confirmed histologically in monkey E. Electrode trajectories were angled parallel to the internal capsule. IC, internal capsule. **(c,d)** Mapping of the STN and SNr in two monkeys. Semi-transparent gray surface plots indicate the edge of STN and SNr nuclei with respect to electrode trajectory, determined electrophysiologically over the course of recording sessions. Each point represents a single stimulation site, and electrical stimulation effects in the spontaneous task are indicated. Different orientations were plotted for Monkey E and Monkey O to maximize the number of visible stimulation sites, because of differences in the recording chamber angle between monkeys. IC, internal capsule.

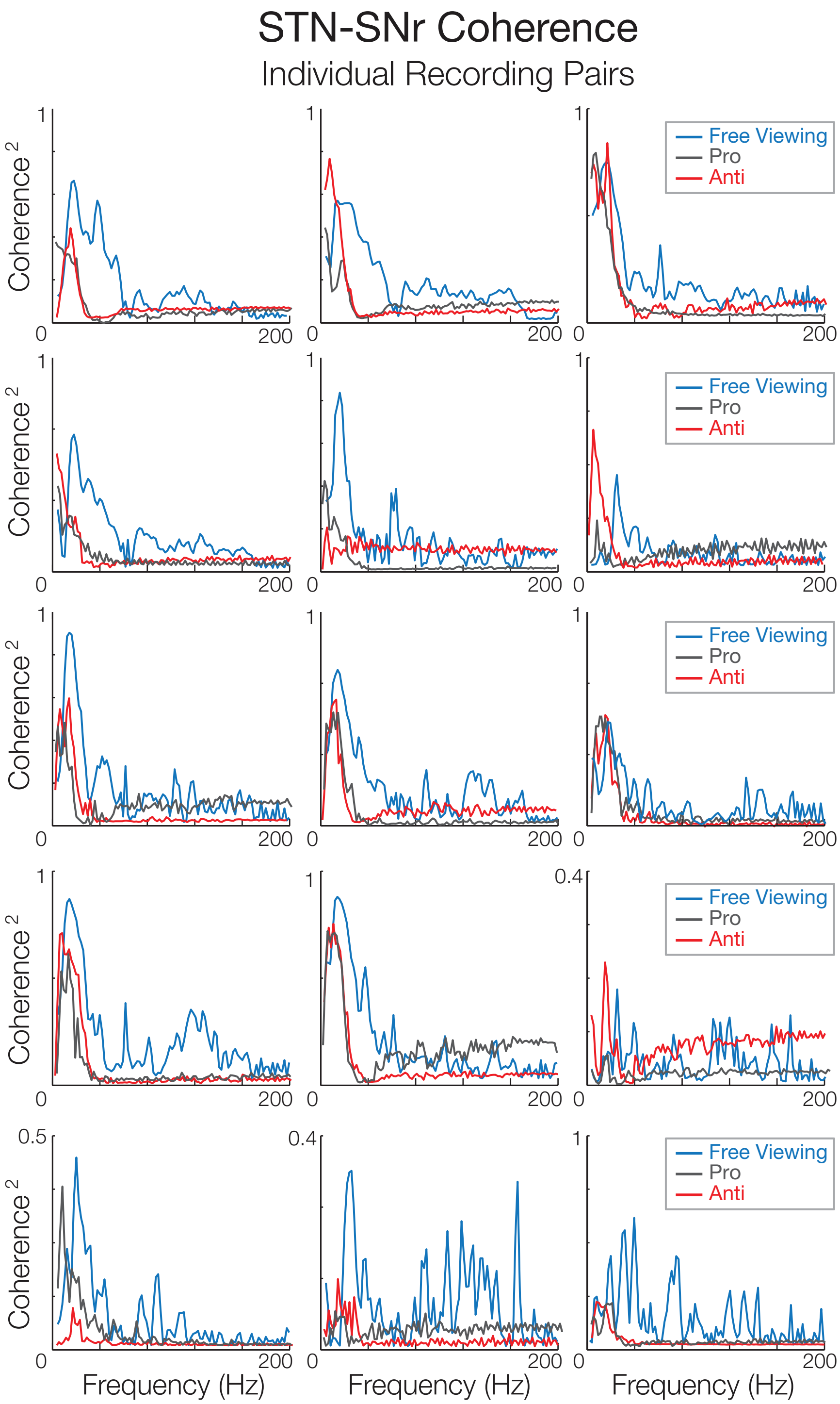

Supplementary Figure 2. Individual STN-SNr pair coherence during unconstrained free viewing, goal-directed anti-saccades, and goal-directed pro-saccades.

Coherence was calculated between simultaneously recorded STN and SNr LFP signals, in the 200 ms period before saccade onset. Coherence is a measure of the variability of the time differences between STN and SNr signals (i.e., phase locking), and approaches 1 if the phase angle between signals is stable and constant over time, or approaches zero if the phase angle

between signals varies greatly. Free viewing saccades were excluded if the monkey did not fixate at least 300 ms before and after saccade onset, to remove contamination from multiple saccade initiations. At each recording site pair, the monkey performed from 500-2000 “correct” free viewing saccade trials (i.e., exhibited appropriate fixation requirements online), and 500-2000 correct interleaved pro- and anti-saccades.

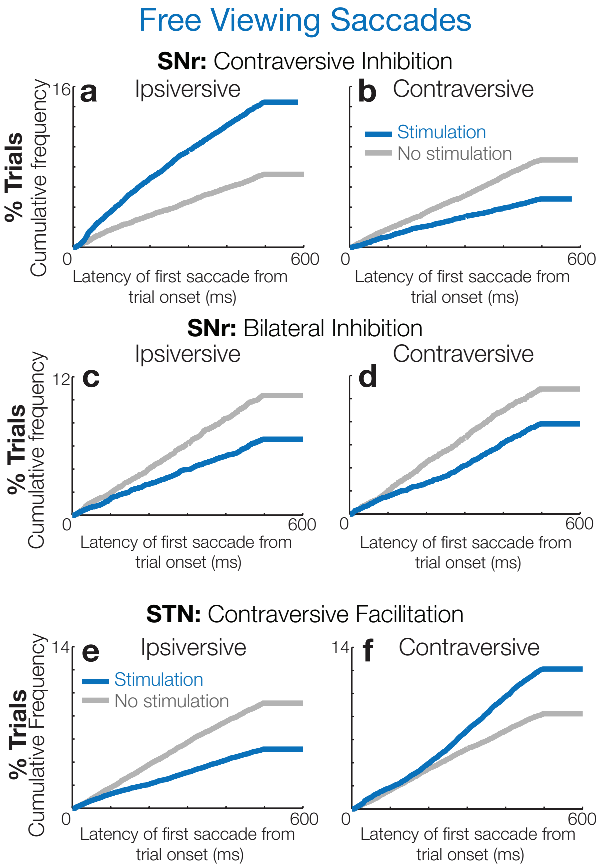

Supplementary Figure 3. Population cumulative distributions of free viewing saccade latencies during SNr and STN electrical stimulation.

SNr stimulation revealed two distinct effects, **(a,b)** saccades were biased toward the ipsiversive visual hemifield (n = 47), and **(c,d)** saccades were inhibited bilaterally (n = 21). The subset of SNr stimulation sites with a bilateral inhibition in saccade latencies (n = 21) was analyzed separately, because there was no appreciable saccade direction bias toward either visual hemifield. **(e,f)** In sharp contrast to the SNr, STN stimulation biased free viewing saccades toward the contraversive visual hemifield (n = 76). The summation of cumulative latencies for saccades toward all directions did not reach 100% because there were trials in which appropriate saccades were not generated within the period of analysis.

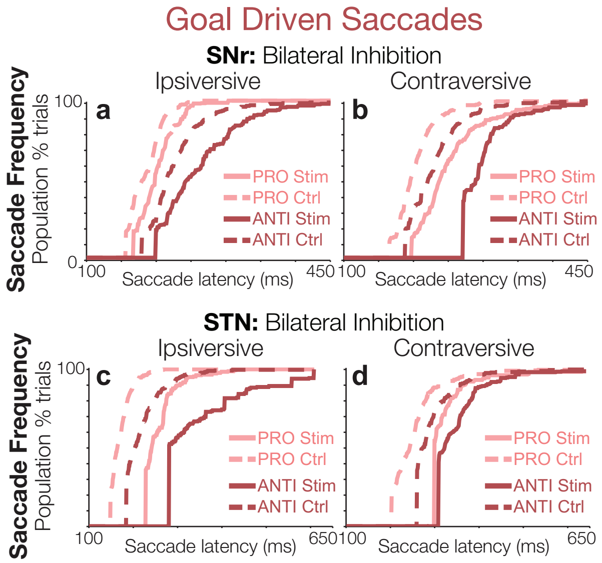

Supplementary Figure 4. Population cumulative distributions of goal driven pro- and anti-saccade latencies during SNr and STN electrical stimulation.

Stimulation of the both the SNr, and the STN during goal driven pro- and anti-saccades inhibited saccades bilaterally. **(a,b)** As in free viewing, SNr stimulation-mediated inhibition was greater on contraversive than ipsiversive saccades, resulting in a bilateral saccade inhibition with a bias toward the ipsiversive visual hemifield overall. **(c,d)** STN stimulation effects were switched between free viewing and goal driven saccade conditions. STN stimulation bilaterally inhibited (not contraversively facilitated) goal driven pro- and anti-saccades.

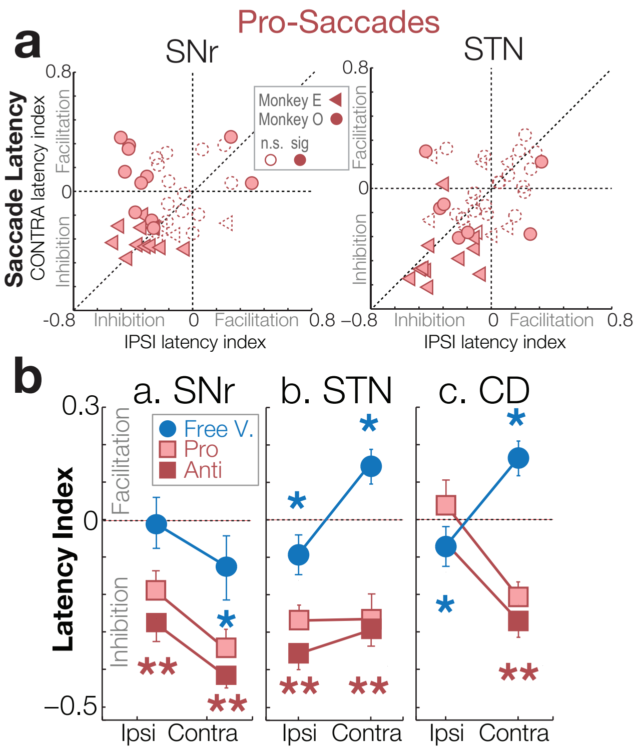

Supplementary Figure 5. STN and SNr pro-saccade stimulation effects, and summary of stimulation effects across all nuclei and task conditions.

(a) Population saccade latency index after SNr or STN stimulation. SNr and STN stimulation both inhibited pro-saccades bilaterally. Each point represents a single stimulation site (120 trials minimum). Filled points are significantly different from 0 (paired *t*-test, $p < 0.05$). n.s., not significant; sig, significant. **(b)** Summary of SNr, STN, and caudate nucleus stimulation effects on saccade latency during free viewing (blue), pro-saccade (hollow red) and anti-saccade (filled red) conditions. Caudate nucleus and STN stimulation resulted in task-specific saccade direction biases while the SNr did not; caudate and STN direction biases were opposite to SNr direction biases during free viewing saccades (i.e., contraversive facilitation versus inhibition), but comparable to the SNr during goal driven pro- and anti-saccades (i.e., bilateral inhibition). In each structure, stimulation effects were qualitatively similar between pro- and anti-saccades when comparing between ipsiversive and contraversive movement directions, but the magnitude of difference in latency index from 0 was larger during anti-saccades.

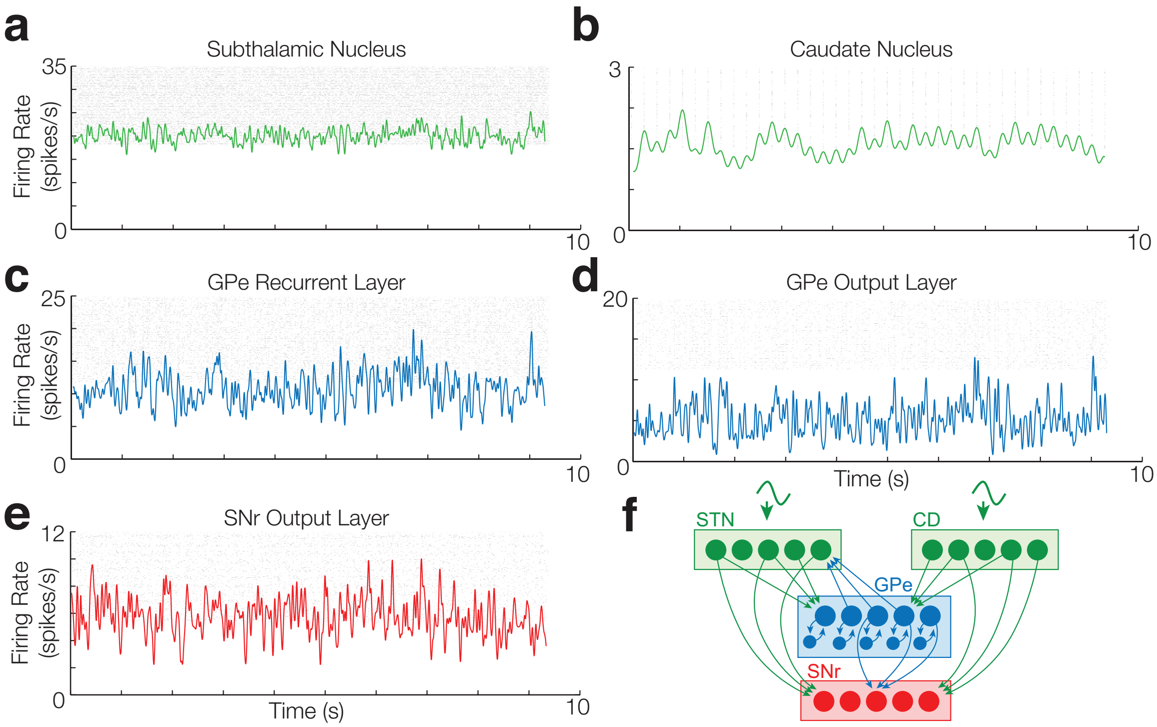

Supplementary Figure 6. Pseudo-real time activity of spiking neural networks basal ganglia model.

A simple spiking neural networks model of the BG was created to test whether altering the input activation of the STN and CD could result in an STN-SNr phase angle shift. Each nucleus ("node") contained 1000 artificial neurons (McCulloch-Pitts neuron model), which projected to other nuclei according to major BG projection pathways. Anatomical localizations of projection neurons within each nucleus were assigned randomly. **(a-e)** Raster and spike density plots of artificial neuron tonic activity sampled from each BG model node. **(f)** Color-coded schematic of BG spiking neural networks model architecture, indicating all modeled projection pathways (hyperdirect, direct, indirect, subthalamo-pallidal-nigral, and recurrent GPe inhibitory layer).

Supplementary Material
II. Tables

Condition		SNr #	SNr %	STN #	STN %
**Free Viewing**	All sites	68	100	76	100
	Ipsiversive bias	18	26	4	5
	Contraversive bias	4	6	35	46
	Bilateral inhibition	21	31	6	8
	Bilateral facilitation	2	3	2	3
**Goal Driven**	All sites	39	100	45	100
	Ipsiversive bias	16	42	7	15
	Contraversive bias	4	10	10	22
	Bilateral inhibition	13	33	12	26
	Bilateral facilitation	2	4	1	2

Supplementary Table 1. Summary of sites with a significant difference in saccade frequency after stimulation (paired *t*-test, $p < 0.05$). Ipsiversive bias describes either increased frequency of ipsiversive saccades, or a greater decrease in contraversive than ipsiversive saccade frequency. Contraversive bias describes either increased frequency of contraversive saccades, or a greater decrease in ipsiversive than contraversive saccade frequency. Bilateral inhibition describes a decrease in both contraversive and ipsiversive saccade frequencies, regardless of an ipsiversive or contraversive bias overall. Each goal driven stimulation site included a minimum of 120 pro-saccade trials + 120 anti-saccade trials (interleaved).
